# SKP-SC-EVs Mitigate Denervated Muscle Atrophy by Inhibiting Oxidative Stress and Inflammation and Improving Microcirculation

**DOI:** 10.3390/antiox11010066

**Published:** 2021-12-28

**Authors:** Wei Wang, Dingding Shen, Lilei Zhang, Yanan Ji, Lai Xu, Zehao Chen, Yuntian Shen, Leilei Gong, Qi Zhang, Mi Shen, Xiaosong Gu, Hualin Sun

**Affiliations:** 1 Key Laboratory of Neuroregeneration of Jiangsu and Ministry of Education, Co-Innovation Center of Neuroregeneration, NMPA Key Laboratory for Research and Evaluation of Tissue Engineering Technology Products, Jiangsu Clinical Medicine Center of Tissue Engineering and Nerve Injury Repair, Nantong University, Nantong 226001, China; ntwangwei911@ntu.edu.cn (W.W.); 1924310007@stmail.ntu.edu.cn (L.Z.); 2022310030@stmail.ntu.edu.cn (Y.J.); xulai@ntu.edu.cn (L.X.); 2022310042@stmail.ntu.edu.cn (Z.C.); syt517@ntu.edu.cn (Y.S.); gonglei@ntu.edu.cn (L.G.); zhangqi@ntu.edu.cn (Q.Z.); will21@ntu.edu.cn (M.S.); nervegu@ntu.edu.cn (X.G.); 2 Department of Pathology, Affiliated Hospital of Nantong University, Nantong 226001, China; 3 Department of Neurology, Shanghai Ruijin Hospital, Affiliated Hospital of Shanghai Jiao Tong University School of Medicine, Shanghai 200025, China; dds@ntu.edu.cn; 4 Nanjing Institute of Tissue Engineering and Regenerative Medicine Technology, Nanjing 210032, China

**Keywords:** denervated muscle atrophy, oxidative stress, inflammation, microcirculation, SKP-SC-EVs

## Abstract

Denervated muscle atrophy is a common clinical disease that has no effective treatments. Our previous studies have found that oxidative stress and inflammation play an important role in the process of denervated muscle atrophy. Extracellular vesicles derived from skin precursor-derived Schwann cells (SKP-SC-EVs) contain a large amount of antioxidants and anti-inflammatory factors. This study explored whether SKP-SC-EVs alleviate denervated muscle atrophy by inhibiting oxidative stress and inflammation. In vitro studies have found that SKP-SC-EVs can be internalized and caught by myoblasts to promote the proliferation and differentiation of myoblasts. Nutrient deprivation can cause myotube atrophy, accompanied by oxidative stress and inflammation. However, SKP-SC-EVs can inhibit oxidative stress and inflammation caused by nutritional deprivation and subsequently relieve myotube atrophy. Moreover, there is a remarkable dose-effect relationship. In vivo studies have found that SKP-SC-EVs can significantly inhibit a denervation-induced decrease in the wet weight ratio and myofiber cross-sectional area of target muscles. Furthermore, SKP-SC-EVs can dramatically inhibit highly expressed Muscle RING Finger 1 and Muscle Atrophy F-box in target muscles under denervation and reduce the degradation of the myotube heavy chain. SKP-SC-EVs may reduce mitochondrial vacuolar degeneration and autophagy in denervated muscles by inhibiting autophagy-related proteins (i.e., PINK1, BNIP3, LC3B, and ATG7). Moreover, SKP-SC-EVs may improve microvessels and blood perfusion in denervated skeletal muscles by enhancing the proliferation of vascular endothelial cells. SKP-SC-EVs can also significantly inhibit the production of reactive oxygen species (ROS) in target muscles after denervation, which indicates that SKP-SC-EVs elicit their role by upregulating Nrf2 and downregulating ROS production-related factors (Nox2 and Nox4). In addition, SKP-SC-EVs can significantly reduce the levels of interleukin 1β, interleukin-6, and tumor necrosis factor α in target muscles. To conclude, SKP-SC-EVs may alleviate the decrease of target muscle blood perfusion and passivate the activities of ubiquitin-proteasome and autophagy-lysosome systems by inhibiting oxidative stress and inflammatory response, then reduce skeletal muscle atrophy caused by denervation. This study not only enriches the molecular regulation mechanism of denervated muscle atrophy, but also provides a scientific basis for SKP-SC-EVs as a protective drug to prevent and treat muscle atrophy.

## 1. Introduction

Skeletal muscle is an important effector organ of the peripheral nervous system, and the structural integrity and functional maintenance are both innervated and controlled by the nervous system [1]. After peripheral nerve injury due to acute trauma, the skeletal muscle can be denervated, and a loss of neural input leads to the subsequent loss of nutrient supply, signal conduction, and material exchange with the nerve. A series of pathological changes, such as reduced muscle fiber cross-sectional area (CSA), destruction of the myofilament sarcomere, decelerated voluntary contraction, and fibrosis, eventually leads to skeletal muscle atrophy and even disability [2]. This impairs quality of life, increases morbidity and mortality, and places heavy burdens on family and society [3]. Because peripheral nerve regeneration is slow, in patients with long-distance nerve defects, irreversible atrophy will appear in the target muscle prior to reinnervation. Therefore, ways by which to delay skeletal muscle atrophy are of great significance to the recovery of target muscle function following nerve repair. However, the treatment of skeletal muscle atrophy remains an unresolved problem, and the molecular mechanism of denervated muscle atrophy has not yet been elucidated. Therefore, a better understanding of the mechanism by which peripheral nerve injury leads to muscle atrophy is crucial; moreover, actively seeking targets for the treatment of muscle atrophy is an important step toward solving a series of problems, such as the recovery of target muscle function after peripheral nerve injury and even the reversal of atrophy.

Oxidative stress and inflammation are two important molecular mechanisms involved in skeletal muscle atrophy [4,5]. Studies have found that when a lack of activity in muscles triggers muscle atrophy, there is an increase in the production of reactive oxygen species (ROS), which significantly increases mitochondrial dysfunction [6,7]; furthermore, ROS play a key role in denervated muscle atrophy [3]. In addition, inflammation is an important pathogenic factor for skeletal muscle dysfunction. Inflammation can damage muscle homeostasis and myogenesis and cause skeletal muscle atrophy [8]. Moreover, excessive interleukin 6 (IL-6) can significantly inhibit mitochondrial function and reduce energy production, thereby causing skeletal muscle atrophy [9]. Our previous studies have found that following peripheral nerve injury resulting from acute trauma, the target muscle is denervated, and this is followed by a series of processes, such as oxidative stress, inflammation, atrophy, and fibrosis, which occur in sequence. Interventions that target these processes can alleviate muscle atrophy and induce a protective effect [4]. Therefore, oxidative stress and inflammation may be potential therapeutic targets to protect against denervated muscle atrophy.

In denervated skeletal muscle, the balance between protein synthesis and protein degradation is disrupted, and excessive protein degradation induces skeletal muscle atrophy [10]. Denervation can activate two main protein degradation pathways: the ubiquitin-proteasome system (UPS) and the autophagy-lysosome system (ALS) by inducing the expression of muscle-specific ubiquitin E3 ligases and autophagy-related proteins in skeletal muscles [3,11,12]. Muscle ring-finger 1 (MuRF1) and muscle atrophy F-box (MAFbx) are two muscle-specific ubiquitin E3 ligases that are most essential to the UPS. They are highly expressed in a variety of muscle atrophy models [13,14,15,16]. Autophagy-related proteins, PINK1, BNIP3, LC3B, and ATG7, are key effectors of the ALS and also play an important role in the process of muscle atrophy. Inhibiting their high expression can delay muscle atrophy [3,17]. In summary, skeletal muscle atrophy is accompanied by the activation of two proteolytic pathways: UPS and ALS.

Various treatments for muscle atrophy have been developed, which primarily include nutritional supplements [18], drug therapy [19], physical therapy [20], electrical stimulation [21,22], and cell therapy [23]. For example, functional Electrical Stimulation represents an effective interventional strategy to attenuate the loss of muscle mass and strength [24]. However, their therapeutic benefits are not adequate in individuals with high severity of muscle injury. Currently, cell therapy is one of the most promising treatment methods. Target cells differentiated from adult stem cells have been used to treat various diseases, and this approach has become a research hotspot. However, adult stem cells are at risk of inducing an immune response and the formation of tumors [23]. Therefore, it is particularly important to develop an alternative therapeutic approach to stem cells.

Extracellular vesicles (EVs) are tiny membrane-containing nanometer- and micrometer-sized vesicles that are secreted into the extracellular space from prokaryotic and eukaryotic cells, such as immune, bone marrow mesenchymal stem, tumor, Schwann, and epithelial cells. EVs mainly include exosomes, microvesicles, and apoptotic bodies [25]. Importantly, EVs contain a variety of biologically active substances, such as cell-signaling molecules (e.g., proteins, lipids, mRNA, miRNA, lncRNA, circRNA, and DNA), which are released into the extracellular space via EVs to influence the behavior of recipient cells. EVs play an extremely important role in intercellular communication, signal transduction, and substance delivery [26,27,28]. Moreover, EVs have an important impact on the physiological and pathological development of the body. The biologically active components of EVs have anti-inflammatory, anti-oxidant, anti-apoptotic, and pro-angiogenesis properties, which have led EVs toward becoming a promising therapeutic tool in the early diagnosis, clinical treatment, and prognostic prediction of diseases [29,30,31,32,33].

Skin-derived precursors (SKPs) are pluripotent stem cells with mesoderm and neural differentiation potential, which are derived from neural crests extracted from the embryonic and adult dermis [34]. Skin-derived precursors pre-differentiated into Schwann cells (SKP-SCs) and have a Schwann cell-like phenotype [35]. In recent years, SKP-SCs have been applied to repair traumatic injury and degenerative diseases of the nervous system, and some progress has been made; therefore, SKP-SCs are now an important tool for neuroregenerative medicine [36,37]. Specifically, SKP-SCs have been shown to improve behavioral recovery in rats with acute and delayed peripheral nerve injury [38]. However, studies have shown that these transplanted cells exert their therapeutic potential primarily through the release of EVs, acting as a paracrine mechanism to enhance endogenous neuroprotection and relieve nerve injury [39]. Therefore, EVs from SKP-SCs (SKP-SC-EVs) may be considered as a promising candidate for the cell-free treatment of nerve injury and regeneration.

We previously confirmed that SKP-SC-EVs promote the axonal growth of the sciatic nerve [40]. A nerve graft constructed from SKP-SC-EVs can repair peripheral nerve defects [41]. In addition, our early RNA sequencing analysis showed that SKP-SC-EVs contain a large number of miRNAs, such as miR-30a-5p, miR-22, miRNA-23a/27a, and miR-27b, which have antioxidant and anti-inflammatory properties [42,43,44,45]. Therefore, we were interested in SKP-SC-EVs as an important mediator of cell-to-cell communication and sought to explore whether, and how, to elicit their role in the treatment of target muscle atrophy after peripheral nerve injury. That is, SKP-SC-EVs may also have significant value in denervated muscle atrophy repair.

This study explored the effects of SKP-SC-EVs on denervated muscle atrophy, analyzed the inhibitory effects of SKP-SC-EVs on oxidative stress and inflammation caused by denervation, investigated the effect of SKP-SC-EVs on target muscle ischemia caused by denervation, and clarified the influence of SKP-SC-EVs on the UPS and ALS pathways during the process of muscle atrophy. This study not only furthered the study of the molecular regulatory mechanism underlying the occurrence and development of denervated muscle atrophy, but also provided new strategies and targets for the clinical treatment of denervated muscle atrophy, which are of great scientific significance and clinical value.

## 2. Materials and Methods

### 2.1. Cell Culture and Treatment

SKPs were isolated from the skin tissues of newborn Sprague-Dawley rats and induced to differentiate into SKP-SCs as described above [40]. SKP-SCs were grown on culture dishes coated with 0.02 mg/mL laminin (Corning Inc., Brooklyn, NY, USA) and 0.2 mg/mL poly-D-lysine (Sigma-Aldrich, St. Louis, MO, USA) within DMEM/F-12 (3:1) (Corning) containing 2% N2 supplement (Stem Cell Technologies, Vancouver, BC, Canada), 50 ng/mL heregulin-1β (R&D, MN, USA), 5 μM forskolin (Sigma-Aldrich, St. Louis, MO, USA), 3% fetal bovine serum (FBS; Gibco, Carlsbad, CA, USA), and 1% penicillin/streptomycin (Beyotime, Shanghai, China). The culture medium was changed every 3–4 days. C2C12 myoblasts were cultured in DMEM (Gibco) medium, supplemented with 10% FBS (Gibco) and 1% penicillin/streptomycin (Beyotime). C2C12 myoblasts were induced to differentiate in medium containing 2% horse serum (American Type Culture Collection, Manassas, VA, USA) for 7 days, and the differentiation medium was changed every 48 h. The differentiated C2C12 myotubes were co-cultured with the vesicles (4 × 10^8^, 8 × 10^8^, 16 × 10^8^, and 32 × 10^8^ particles/mL), which were dissolved in amino acid- and serum-free Hank’s balanced salt solution (HBSS; Gibco). After 12 h of co-incubation, the C2C12 myotubes were subjected to morphological measurement, biochemical detection, and RNA or protein extraction for subsequent experiments. In addition, human umbilical vein endothelial cells (HUVECs) were cultured with endothelial cell medium (ScienCell 1001, Beijing, China) supplemented with 5% FBS (Gibco), 1% penicillin/streptomycin (Beyotime), and ECGS (ScienCell 1052, Beijing, China). The fused cells were used for subsequent experiments.

### 2.2. Animals

All animal experiments followed the guidelines of Nantong University’s animal care standards and were approved by the Jiangsu Provincial Laboratory Animal Management Committee (ethical code: S20200312-003). ICR mice (male, body mass 18–20 g) provided by the Experimental Animal Center of Nantong University, were randomly divided into three different groups. Mice underwent left sciatic nerve dissection. SKP-SC-EVs were resuspended in 20 μL of PBS buffer, followed by multi-site injections 5 × 10^10^ EV particles (Den + EV group) or PBS (Den + PBS group) into the tibialis anterior muscle using a micro-syringe. Mice in the normal control group underwent a sham operation (sham group) and were injected with the same amount of PBS. The wounds were sutured as routine and disinfected with iodophor. All mice were fed normally at room temperature (20 ± 2 °C) with free access to water and food. After 14 days, the mice were anesthetized, killed by cervical decapitation and the tibialis anterior muscle was taken, weighed, frozen in liquid nitrogen, and then stored at −80 °C.

### 2.3. Isolation and Identification of Vesicles

The vesicles were isolated and identified as per the MISEV 2018 guidelines [46]. First, the EVs were released by the SKP-SCs. SKP-SCs at 80–90% confluency were incubated in a proliferation medium without FBS for 48 h. The harvested conditioned medium was centrifuged at 500× *g* for 10 min. The supernatant was collected and filtered through a 0.22 μm filter (Millipore). The filtrate was subjected to gradient centrifugations at 300× *g* for 10 min, 2000× *g* for 10 min, and 10,000× *g* for 30 min at 4 °C. The supernatant was retained, and the cell residue was discarded. The supernatant collected was ultracentrifuged at 100,000× *g* for 90 min at 4 °C. After removal of the supernatant, the concentration and size distribution of the precipitated SKP-SC-EVs were measured using nanoparticle tracking analysis (NTA, Particle Metrix, Germany). The morphology and size of EVs were observed under a transmission electron microscope (HT7700, Hitachi, Tokyo, Japan). Western blot assay was used to detect EV-labeled proteins, CD9, CD63, and CD81, and heat shock protein 70 (HSP70), and thus the precipitates were confirmed to be EVs, and used for subsequent experiments.

### 2.4. Internalization of PKH67-Labelled SKP-SC-EVs

EVs were fluorescently labeled per the protocol described above [47]. First, PKH67-labeled EVs were internalized by cultured myoblasts and subsequently subjected to immunofluorescence staining and observation. Briefly, SKP-SC-EVs were dyed with the PKH67 Green Fluorescent Linker Mini Kit (Sigma-Aldrich, St. Louis, MO, USA), and the EVs were then suspended in Amicon Ultra 10 kDa tubes (Millipore, Billerica, MA, USA) and concentrated. The C2C12 myoblasts were then incubated with PKH67-labeled EVs or PKH67 controls for 4 h and then subjected to Desmin (1:400, Abcam) immunofluorescence staining. Images were acquired using a fluorescence microscope (Zeiss, Oberkochen, Germany). For in vivo internalization, SKP-SC-EVs were labeled with PKH67 at 37 °C. Subsequently, 20 μL of PBS containing SKP-SC-EVs particles were microinjected into the tibialis anterior muscle. PBS incubated with PKH67 were microinjected into the tibialis anterior muscle as the control. Twenty-four hours after the injection, the tibialis anterior muscle was separated and sliced into frozen 10-μm thick sections, immunofluorescently stained with laminin (1:1000, Sigma-Aldrich), labeled with 4′,6-diamidino-2-phenylindole (DAPI), and photographed using a fluorescence microscope.

### 2.5. Immunofluorescence Staining

Immunofluorescence staining of SKP-SCs was performed to identify the expression of cellular protein markers. The cell samples were fixed with 4% paraformaldehyde (Beyotime, Shanghai, China) for 15 min at room temperature and rinsed thrice with PBS. Cells were blocked with a blocking solution (Beyotime, Shanghai, China) at room temperature for 1 h and incubated with the primary antibody overnight at 4 °C and with the second antibody in the dark for 2 h. The primary antibodies included rabbit anti-S100β (1:400, Invitrogen) and chicken-anti-glial fibrillary acidic protein (anti-GFAP, 1:1000, Abcam). The cells were finally co-stained with DAPI (Sigma-Aldrich), and photographed with a fluorescence microscope.

The diameter of the myotube was detected using myotube heavy chain (MHC) staining. C2C12 cells grew and differentiated into myotubes. The C2C12 myotubes were then fixed with 4% paraformaldehyde (Beyotime, Shanghai, China), blocked with a blocking solution (Beyotime) for 1 h, and incubated with mouse anti-MHC antibody overnight at 4 °C (1:200, R&D Systems). After rinsing with PBS, the myotubes were incubated with myeloperoxidase Alexa Fluor 594 anti-mouse (1:400, Thermo Fisher, Waltham, MA, USA) at room temperature for 30 min. The immunostained myotubes were observed under a fluorescence microscope. All multinucleated cells that were positive for MHC and contained at least three nuclei were defined as myotubes [48]. We then calculated the size of the myotubes. At least 100 myotubes were taken for diameter calculation using the Image J software 1.8.0 (National Institutes of Health, Bethesda, MD, USA) under each condition. Three measurements along the myotube length were averaged to obtain a mean diameter for the myotubes.

### 2.6. Immunohistochemical Analysis

To analyze the delaying effect of SKP-SC-EVs on muscle atrophy in vivo, 14 days after treatment with SKP-SC-EVs, the tibialis anterior muscle of the mice was dissected under anesthesia and fixed with 4% paraformaldehyde (Beyotime, Shanghai, China) for 12 h, followed by sucrose gradient dehydration. Subsequently, frozen 10-μm thick sections were cut using a cryostat (CM3050 S, Leica, Mannheim, Germany), and the sections were placed in a 37 °C oven overnight and rinsed thrice in PBS for 5 min each. After an appropriate amount of blocking solution (Beyotime, Shanghai, China) was added, the sections were blocked at 37 °C for 1 h, incubated with the primary antibody at 4 °C overnight, immunofluorescently stained with mouse anti-laminin (1:1000, Abcam, Cambridge, UK) and goat anti-CD31 (1:200, Cell Signaling, Cambridge, UK) at 4 °C overnight, and incubated with the secondary antibody Alexa Fluor 594-goat anti-rabbit immunoglobulin G (IgG; 1:600, Abcam) at room temperature in the dark for 2 h. The sections were mounted with DAPI mounting solution. The tibial anterior muscle was photographed using the fluorescence microscope, and the CSA of muscle fibers in each field of view was measured using the ImageJ software. Differences in the CSA of muscle fibers among groups were analyzed statistically. Vascular fluorescence intensity of the muscle fibers per field of view was measured and analyzed statistically.

### 2.7. Transmission Electron Microscopy Analysis

Muscle observation under a transmission electron microscope has been reported in detail previously [49]. Under anesthesia, a 1 mm^3^ sample of the tibialis anterior muscle was taken and fixed in ice cold 2.5% glutaraldehyde for 24 h and then fixed in 1% osmium tetroxide for 2 h. The sample was quickly dehydrated in a concentration gradient ethanol (30–100%) solution, immersed in 100% acetone, and embedded in a low-viscosity epoxy resin. Muscle samples were sectioned and stained on a grid with 1% uranyl acetate and lead citrate and observed under a transmission electron microscope (HT7700, Hitachi, Tokyo, Japan).

### 2.8. ROS Detection

The total ROS level in the C2C12 myotube or tibialis anterior muscle was detected using a dichlorodihydrofluorescein diacetate (DCFH-DA) or dihydroethidium (DHE) fluorescent probe. Briefly, C2C12 myotubes were washed with PBS and fresh DMEM and incubated with 10 μM DCFH-DA for 20 min in the dark at room temperature. When the excitation wavelength was 488 nm and the emission wavelength was 519 nm, the production of ROS was detected using dichlorofluorescein (DCF) fluorescence. Animals were perfused with DHE (10 μM), and the tibialis anterior muscle was prepared for DHE staining. The tibialis anterior muscle sample was obtained and sectioned, followed by immunofluorescence staining under a fluorescence microscope. The fluorescence intensity value obtained was normalized to the untreated control value.

### 2.9. Cell Proliferation Analysis

The EdU Cell Proliferation Assay Kit (RiboBio, Guangzhou, China) was used to measure cell proliferation. C2C12 myoblasts or HUVECs, which were cultured in the conditioned medium with different concentrations of EVs or SKP-SCs, were incubated in a fresh medium containing 50 μM 5-ethynyl-2′-deoxyuridine (EdU) and cultured in a cell incubator at 37 °C for 2 h. After rinsing in PBS, the cells were fixed in 4% paraformaldehyde for 30 min and treated with 0.5% Triton X-100 for 10 min. Hoechst 3342 was used for staining the nuclei for 15 min. Finally, the proportion of C2C12 myoblasts or HUVECs that proliferated in the culture medium containing EdU was counted.

### 2.10. Quantitative Reverse Transcriptase-Polymerase Chain Reaction

As indicated by manufacturer instructions, the TRIzol reagent (QIAGEN) was used to extract the total RNA from cell lines and tissues, and the Omniscript RT Kit (QIAGEN) was used to synthesize cDNAs. The SYBR Green Master Mix Kit (Lithuania) and the BIO-RAD system (BIO-RAD-96CFX) were used for real-time fluorescence quantitative PCR (qPCR) detection. The qPCR conditions were as follows: 95 °C for 10 min; then 40 cycles of 10 s at 95 °C, 30 s at 60 °C, and 10 s at 72 °C; followed by a melting curve analysis from 65 °C to 95°C. The small subunit ribosomal RNA (18sRNA) was used as the mRNA standard. All primers used in this study are listed in Table 1. The relative mRNA expression was detected using 2^−ΔΔCt^ method [50].

### 2.11. Western Blot Analysis

Western blot assay was performed as previously reported [3]. Pierce RIPA lysis buffer (Thermo Fisher Scientific, Waltham, MA, USA) containing protease and phosphatase inhibitors (CST, Danvers, MA, USA) was used to extract protein samples from the skeletal muscle or different groups of cells. The supernatant was centrifuged at 12,000 rpm for 30 min at 4 °C. The protein concentration was determined using the BCA assay kit (Thermo Fisher Scientific, Waltham, MA, USA). The same amount of total protein was separated with 12% sodium dodecyl sulfate-polyacrylamide gel electrophoresis, transferred to a polyvinylidenedifluoride membrane (Millipore, MA, USA), blocked with Tris buffer saline containing 5% skimmed milk powder, followed by incubation with primary antibodies overnight at 4 °C. The primary antibodies included: anti-MHC polyclonal antibody (T421/S424) (1:1000, R&D System), anti-MuRF1 polyclonal antibody (1:1000, Abcam), anti-MAFbx polyclonal antibody (1:1000, Abcam), anti-Nrf2 polyclonal antibody (1:1000, Abcam), anti-Nox2 polyclonal antibody (1:1000, Abcam), anti-Nox4 polyclonal antibody (1:1000, Abcam), anti-BNIP3 polyclonal antibody (1:1000, Abcam), anti-LC3B polyclonal antibody (1:1000, MBL), anti-PINK1 polyclonal antibody (1:1000, Abcam), anti-ATG7 polyclonal antibody (1:1000, Abcam) and anti-beta tubulin polyclonal antibody (1:1000, Abcam). After rinsing 3 times in 1 × tris-buffered saline with 0.1% Tween 20 detergent, the membranes were incubated with horseradish peroxidase-labeled goat anti-mouse IgG (1:5000, Santa Cruz Biotechnology, Dallas, TX, USA) and horseradish peroxidase-labeled chicken anti-rabbit IgG (1:5000, Abcam, Cambridge, MA, USA) at room temperature for 2 h. The immunoreactive bands were detected using enhanced chemiluminescence reagent kits (Thermo Scientific, Park Ellisville, MO, USA). The intensity of the bands was analyzed using the ImageJ software and further normalized with the loading control. In addition, a western blot assay was used to detect SKP-SC-EVs- and SKP-SCs-related marker proteins. The primary antibodies used included anti-CD9 (1:1000, Abcam), anti-CD63 (1:1000, Abcam), anti-CD81 (1:1000, Abcam), anti-HSP70 (1:1000, Proteintech, Rosemont, IL), anti-Calnexin (1:1000, Abcam), β-actin (1:2000, Abcam), and anti-S100β (1:400, Invitrogen).

### 2.12. Ultrasound Imaging Analysis

The mice were anesthetized, and the blood flow of the hind limbs was monitored using a laser Doppler perfusion imager (PeriScan PIM 3 System, Perimed AB, Stockholm, Sweden) on the 3rd, 7th, and 14th days after the operation. On the ultrasound image, the perfusion ratio was indicated as the ratio of the ischemic area on the surgical side to the non-ischemic area on the contralateral side.

### 2.13. Statistical Analysis

All data are expressed as means ± standard errors of the mean and analyzed using a one-way analysis of variance. Intergroup differences were detected using Tukey’s multiple comparisons test. All statistical analyses were performed using the GraphPad Prism software (version 7; San Diego, CA, USA). A value of *p* < 0.05 was considered statistically significant.

## 3. Results

### 3.1. Identification of SKP-SCs

We resuscitated SKP-SCs at passages 14–16, which had been purified and cryopreserved, and the cells were cultured in a low-serum differentiation medium containing Forskolin, HRG, and N2. Our findings were consistent with the results of previous studies. Under a phase-contrast microscope, typical bipolar or tripolar Schwann cell-like cells were long spindle-shaped and scattered, with a strong refractive index. After subculturing, SKP-SCs were arranged closely along the longitudinal axis and had grown side by side (Supplementary Figure S1A). S-100β and GFAP immunofluorescence chemical staining indicated that SKP-SCs were positive for SC-specific markers, GFAP and S-100β (Supplementary Figure S1B), and the average purity of SKP-SCs was 98% (S-100β positive percentage). Therefore, these high-purity SKP-SCs could be used for subsequent experiments.

### 3.2. Isolation and Identification of SKP-SC-EVs

**Figure 1:**
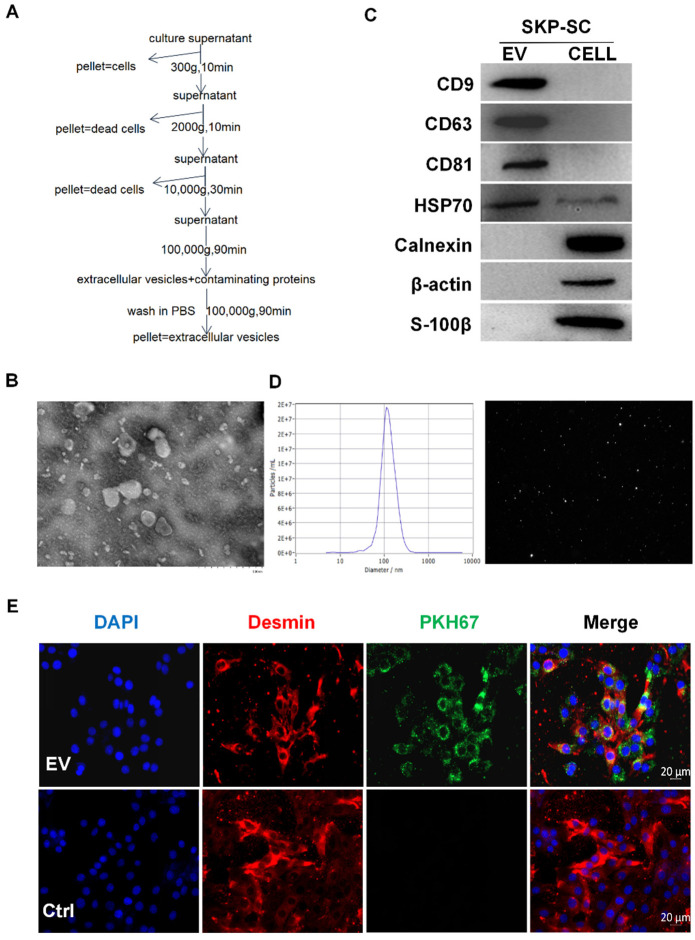
Identification of EVs from SKP-SCs. (**A**) Schematic diagram of gradient ultracentrifugation steps for separating and extracting EVs derived from SKP-SCs. (**B**) Representative transmission electron microscope images of SKP-SC-EVs. Scale bar = 500 nm. (**C**) Western blot analysis shows the positive expression of exosomal markers CD9, CD63, CD81, and HSP70 in EVs. Calnexin, β-actin, and S-100β in SKP-SCs acted as control markers positively expressed in SKP-SCs. (**D**) NTA of representative SKP-SC-EV particles. (**E**) Representative SKP-SC-EVs labeled with PKH67 (green) in the cytoplasm of C2C12 myoblasts (red label indicates myosin), and the nucleus labeled with DAPI (blue). Scale bar = 20 μm. EVs, extracellular vesicles; SCs, Schwann cells; SKP, skin-derived precursors.

SKP-SCs were starved, and the supernatant was collected and ultracentrifuged to extract EVs (Figure 1A). We identified SKP-SC-EVs by assessing the morphology, particle size, concentration, and marker protein expression of the EVs. Under a transmission electron microscope, most EVs exhibited a spherical double-layer membrane structure with a cup-shaped concave cavity (Figure 1B). Western blot assay indicated that the positive markers of EVs, including CD9, CD63, CD81, and HSP70, were also expressed in SKP-SC-EVs. Compared with the released EVs, SKP-SCs displayed negative expression of CD9, CD63, and CD81, whereas the expression of HSP70 was lower. Calnexin, β-actin, and S-100β, which were expressed in SKP-SCs, were used as control markers for quality control (Figure 1C). The NTA results showed that the average diameter of EVs was 117.8 nm, the particle size peaked at 183.7 nm, and the concentration was approximately 7.1 × 10^11^ particles/mL (Figure 1D). These findings collectively indicated that the SKP-SC-EVs were successfully obtained. In addition, we identified the activity of SKP-SC-EVs by co-culturing EVs with C2C12 myotubes under hypoxia and found that SKP-SC-EVs can protect against myotube atrophy under hypoxia (Supplementary Figure S2). These findings confirmed that we obtained active SKP-SC-EVs. We also investigated whether SKP-SC-EVs can be transferred to and interact with C2C12 myoblasts. After C2C12 myoblasts were incubated with PKH67-labeled SKP-SC-EVs in vitro for 4 h, the immunostaining image showed that C2C12 took up the PKH67-labeled EVs, and a PKH67-positive signal was detected in the cytoplasm of C2C12 (Figure 1E). This indicated that SKP-SC-EVs can be internalized and taken up by C2C12 and that an interaction may exist between them.

### 3.3. SKP-SC-EVs Promote C2C12 Proliferation and Differentiation

**Figure 2:**
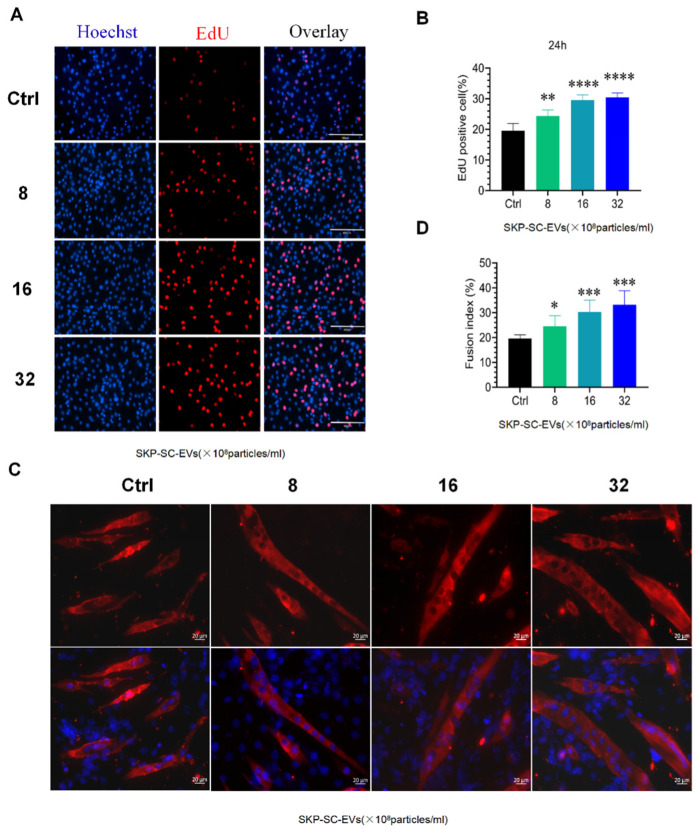
SKP-SC-EVs promote C2C12 cell proliferation and differentiation. (**A**) Representative EdU staining image of C2C12 cells 12 h after SKP-SC-EV or control treatment. Scale bar = 400 μm. (**B**) Histogram indicating the number of proliferating cells in the EV and control (Ctrl) groups. *n* = 4, ** *p* < 0.01 and **** *p* < 0.0001 vs. Ctrl group. (**C**) SKP-SC-EV effects on the induction of C2C12 cell fusion. Scale bar = 20 μm. (**D**) Histogram indicating the effect of different doses of SKP-SC-EVs on the myotube fusion index. *n* = 4, * *p* < 0.05 and *** *p* < 0.001 vs. Ctrl group. EVs, extracellular vesicles. SCs, Schwann cells; SKP, skin-derived precursors.

To explore whether SKP-SC-EVs influence the biological behavior of C2C12 cells, we first examined the effects of different concentrations of SKP-SC-EVs on the proliferation of C2C12 cells through the Transwell chamber device, in which SKP-SC-EVs and C2C12 cells could be separated. The EdU analysis showed that compared with the control group, SKP-SC-EV treatment promoted the proliferation of C2C12 cells significantly more after 24 h of co-culturing. When the concentration of EVs changed from 8 × 10^8^ to 32 × 10^8^ particles/mL, the proliferation ability was significantly increased in a dose-dependent manner, and cell proliferation was significantly higher in the high-dose group than in the low-dose group, which indicated that SKP-SC-EVs stimulate the proliferation of C2C12 cells in vitro (Figure 2A,B). Subsequently, we further observed the effect of SKP-SC-EVs on the differentiation of C2C12 cells. After co-culturing, SKP-SC-EVs significantly promoted the differentiation of C2C12 cells into multinucleated myotubes in a dose-dependent manner. As the concentration of SKP-SC-EVs increased, the number of multinucleated myotubes increased significantly (Figure 2C,D). Thus, when SKP-SC-EVs are taken into C2C12 cells, SKP-SC-EVs significantly promote the proliferation and differentiation of C2C12 cells in a dose-dependent manner.

### 3.4. SKP-SC-EVs Mitigate C2C12 Myotube Atrophy Caused by Nutrient Deprivation

**Figure 3:**
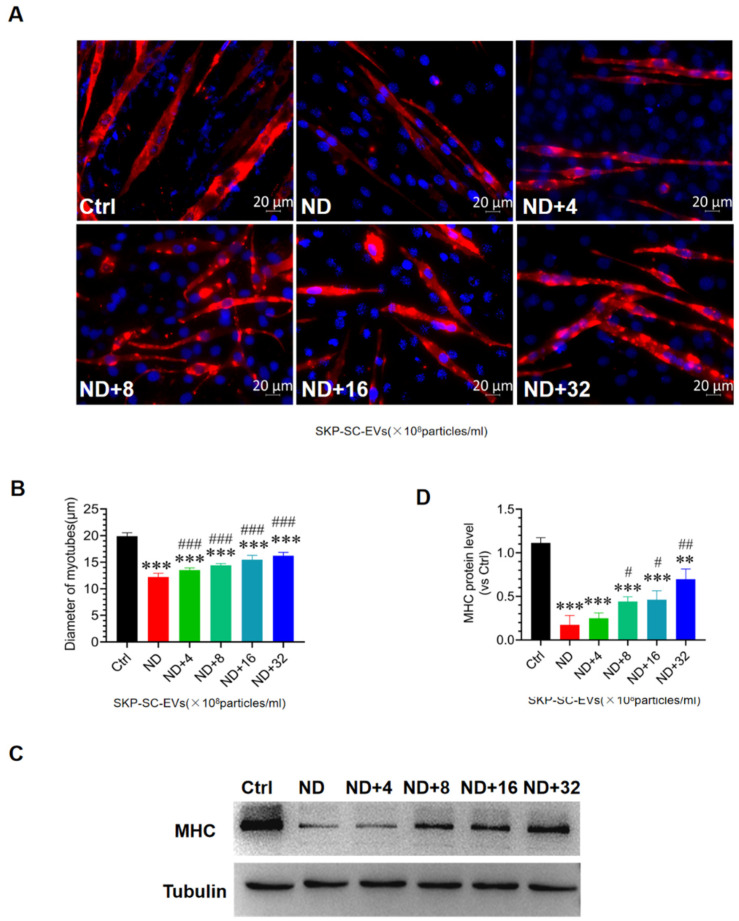
SKP-SC-EVs relieve C2C12 myotube atrophy caused by nutrient deprivation. (**A**) Myosin heavy chain (MHC) immunofluorescence image of myotubes 12 h after treatment with HBSS with or without SKP-SC-EVs (4 × 10^8^, 8 × 10^8^, 16 × 10^8^, and 32 × 10^8^ particles/mL). Red represents MHC staining. Scale bar = 20 μm. (**B**) Measurement of myotube diameter. After SKP-SC-EV treatment, the diameter of myotubes gradually increased. *n* = 4, *** *p* < 0.001 vs. Ctrl group, ### *p* < 0.001 vs. ND group. (**C**) Western blot images of MHC expression in different treatment groups. (**D**) Representative western blot detection of changes in MHC expression. *n* = 3, ** *p* < 0.01 and *** *p* < 0.001 vs. Ctrl group; # *p* < 0.05 and ## *p* < 0.01 vs. ND group. Ctrl, control; EVs, extracellular vesicles; HBSS, Hank’s balanced salt solution; ND, nutritional deprivation; SCs, Schwann cells; SKP, skin-derived precursors.

To determine whether SKP-SC-EVs can alleviate C2C12 myotube atrophy, we established a C2C12 myotube atrophy model following nutrient deprivation. C2C12 myotubes were incubated in HBSS with or without SKP-SC-EVs (4 × 10^8^, 8 × 10^8^, 16 × 10^8^, 32 × 10^8^ particles/mL) for 12 h, and C2C12 myotubes were then stained with MHC fluorescence. Compared with the control group, 12 h of nutrition deprivation induced significantly greater C2C12 myotube atrophy, and the diameter of the myotube was lower in the nutrition deprivation group. Interestingly, SKP-SC-EVs reduced myotube atrophy caused by nutrient deprivation. After treatment with EVs at a concentration of 4 × 10^8^ particles/mL, the myotube atrophy phenotype was significantly reversed, and the diameter of the myotube was increased. After the addition of SKP-SC-EVs, the diameter of the myotube showed a dose-dependent increase (Figure 3A,B). High-dose SKP-SC-EVs (32 × 10^8^ particles/mL) had better performance in reducing myotube atrophy. The western blot assay further indicated that compared with the control group, the MHC expression level decreased more in the nutrition deprivation group, and compared with the nutrition deprivation group, MHC expression showed a significantly greater dose-dependent increase after treatment with EVs at different concentrations. As the concentration of EVs increased, the expression of MHC gradually increased (Figure 3C,D). Taken together, these findings indicate that the addition of SKP-SC-EVs alleviates C2C12 myotube atrophy after nutrient deprivation.

### 3.5. SKP-SC-EVs Inhibit Nutrient Deprivation-Induced Oxidative Stress and Inflammation in C2C12 Myotubes

**Figure 4:**
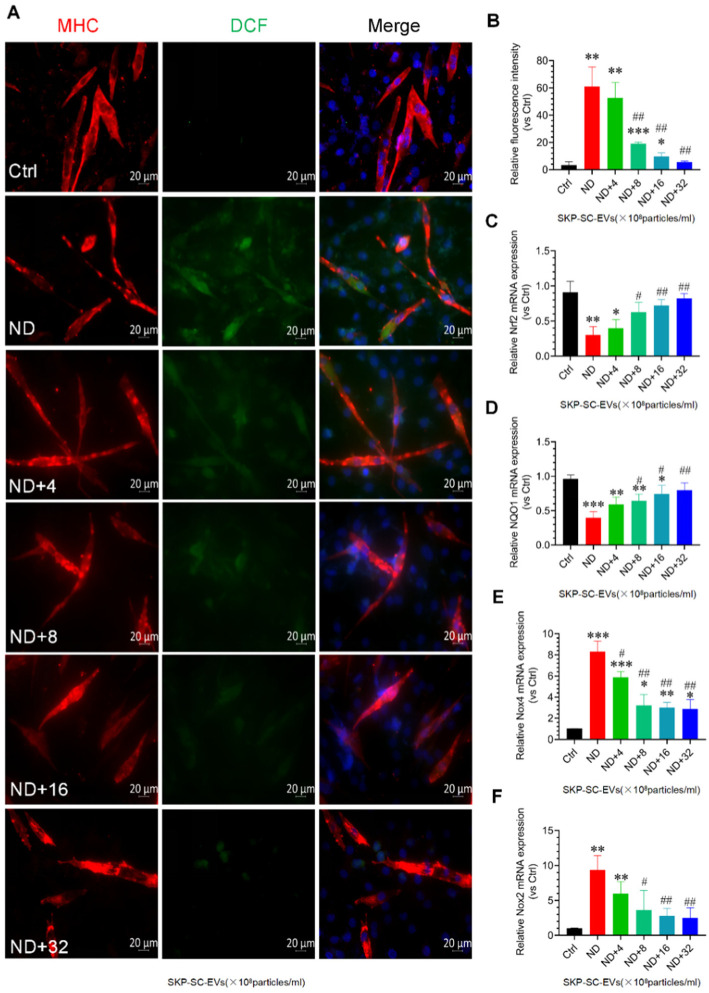
SKP-SC-EVs inhibit oxidative stress of myotubes after nutrient deprivation. (**A**) DCF staining images of myotubes 12 h after treatment with HBSS with or without SKP-SC-EVs (4 × 10^8^, 8 × 10^8^, 16 × 10^8^, and 32 × 10^8^ particles/mL). Red and green represent MHC staining and DCF staining, respectively. Blue fluorescence in far right panels represent DAPI-stained nuclei. Scale bar = 20 μm. (**B**) Histogram indicating that SKP-SC-EVs inhibit oxidative stress of atrophic myotubes after nutrient deprivation. *n* = 4, * *p* < 0.05, ** *p* < 0.01, and *** *p* < 0.001 vs. Ctrl group; ## *p* < 0.01 vs. ND group. (**C**–**F**) qRT-PCR analysis of oxidative stress-related genes Nrf2, NQO1, Nox4, and Nox2 in myotubes. *n* = 3, * *p* < 0.05, ** *p* < 0.01, and *** *p* < 0.001 vs. Ctrl group; # *p* < 0.05 and ## *p* < 0.01 vs. ND group. Ctrl, control; DCF, dichlorofluorescein; EVs, extracellular vesicles; HBSS, Hank’s balanced salt solution; ND, nutritional deprivation; SCs, Schwann cells; SKP, skin-derived precursors.

**Figure 5:**
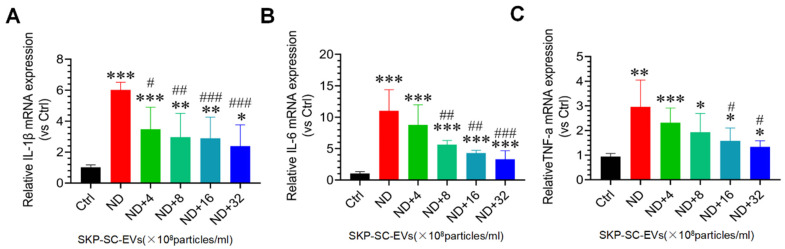
SKP-SC-EVs inhibit inflammation in myotubes after nutrient deprivation. (**A**–**C**) qRT-PCR analysis of the expression of IL-1β, IL-6, and TNF-α in myotubes 12 h after treatment with HBSS with or without SKP-SC-EVs (4 × 10^8^, 8 × 10^8^, 16 × 10^8^, and 32 × 10^8^ particles/mL). *n* = 3, * *p* < 0.05, ** *p* < 0.01, and *** *p* < 0.001 vs. Ctrl group; # *p* < 0.05, ## *p* < 0.01, and ### *p* < 0.001 vs. ND group. Ctrl, control; DCF, dichlorofluorescein; EVs, extracellular vesicles; HBSS, Hank’s balanced salt solution; IL, interleukin; ND, nutritional deprivation; SCs, Schwann cells; SKP, skin-derived precursors; TNF-α, tumor necrosis factor-α.

Oxidative stress and inflammation are two important molecular mechanisms involved in skeletal muscle atrophy [4,5]. In this study, C2C12 myotubes treated with HBSS significantly induced ROS production, and strong DCF was observed in the myotubes under fluorescence microscopy. Interestingly, SKP-SC-EVs treatment inhibited the production of ROS in C2C12 myotubes treated with HBSS, as confirmed by a significant reduction in DCF fluorescence. In addition, high concentrations of SKP-SC-EVs showed better performance in inhibiting the production of ROS (Figure 4A,B). We also found that in C2C12 myotubes treated with HBSS, Nox2 and Nox4 mRNA expression was significantly increased, whereas Nrf2 and NQO1 mRNA expression was significantly inhibited. As the concentration of SKP-SC-EVs increased, SKP-SC-EVs reversed these responses following nutrient deprivation (Figure 4C–F). Our study also indicated that myotube atrophy induced by nutrient deprivation significantly induces the expression of marker of inflammation, as evidenced by an increase in the expression levels of inflammatory cytokines, IL-1β, IL-6, and TNF-α. SKP-SC-EVs can reduce the expression of inflammatory cytokines in a dose-dependent manner, and high-dose SKP-SC-EVs produced a better anti-inflammatory effect (Figure 5). All of these findings suggest that SKP-SC-EVs inhibit oxidative stress and inflammation in C2C12 myotubes caused by nutrient deprivation.

### 3.6. SKP-SC-EVs Relieve Skeletal Muscle Atrophy Caused by Denervation

**Figure 6:**
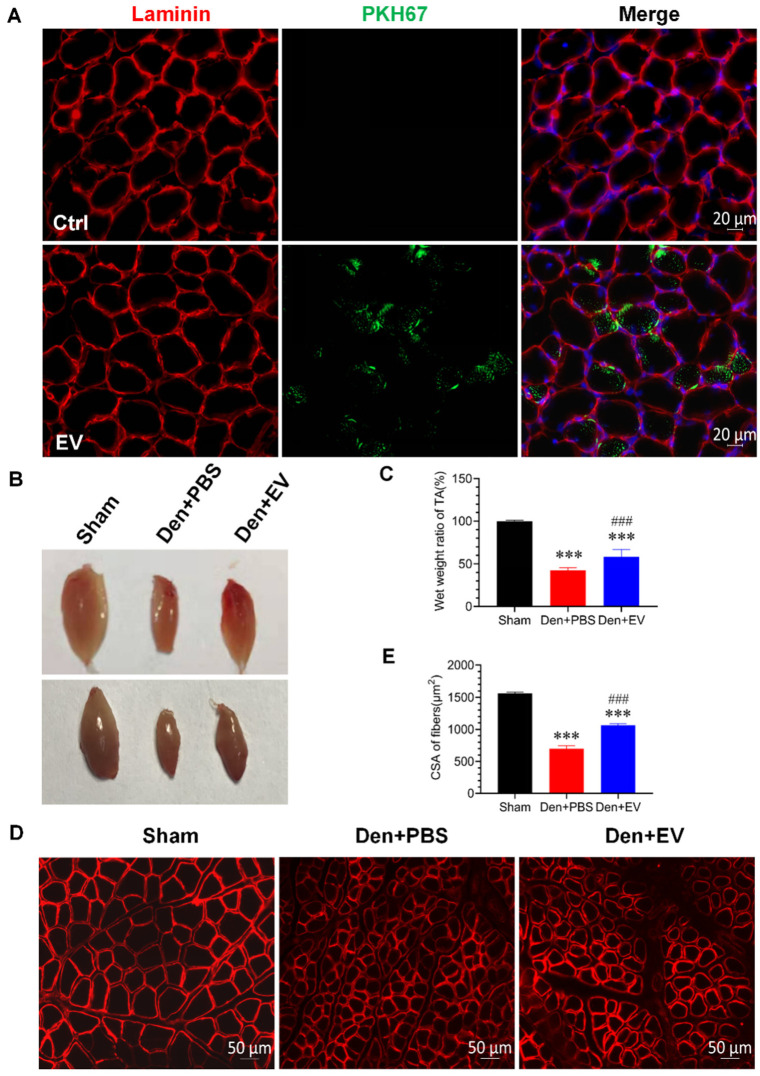
SKP-SC-EVs alleviate denervated skeletal muscle atrophy. (**A**) EVs labeled with PKH67 (green) are displayed in the muscle fibers (red), and the nucleus is labeled with DAPI (blue). Scale bar = 20 μm. The tibialis anterior muscle was injected with PBS (Ctrl group) or PKH67-labeled EVs (EV group). (**B**) General observation of the effect of SKP-SC-EVs on denervated muscle atrophy. (**C**) Histogram indicating the effect of SKP-SC-EVs on the wet weight ratio after denervated muscle atrophy. *n* = 10, *** *p* < 0.001 vs. Sham group; ### *p* < 0.001 vs. Den + PBS group. (**D**) Laminin (red) staining of muscle fiber cross-sections in different treatment groups after denervated muscle atrophy. Scale bar = 50 μm. (**E**) Statistical analysis of muscle fiber cross-sections. *n* = 5, *** *p* < 0.001 vs. Sham group; ### *p* < 0.001 vs. Den + PBS group. The sham group was injected with PBS; the Den group was injected with PBS containing SKP-SC-EVs (5 × 10^10^ particles) (Den + EV group), and; the Ctrl PBS group (Den + PBS group) was injected with PBS. The tibialis anterior muscle samples were collected 14 days after treatment. Den, denervation; EVs, extracellular vesicles; SCs, Schwann cells; SKP, skin-derived precursors.

In this study, we used an in vivo model to further confirm that SKP-SC-EVs can alleviate denervation-induced skeletal muscle atrophy. Before evaluating the effects of SKP-SC-EVs on denervated skeletal muscle atrophy, we investigated whether SKP-SC-EVs can be internalized by skeletal muscles. PKH67-labeled EVs were microinjected into skeletal muscles. Twenty-four hours later, the muscle samples on the injected side were collected, frozen, and cut into sections, followed by laminin immunofluorescence staining. Green EV particles were widely distributed on the cross-section of the muscle tissue, which indicated that SKP-SC-EVs labeled with PKH67 can be internalized and taken up by skeletal muscles (Figure 6A). Subsequently, an intramuscular injection was administered to treat the mouse model of denervated muscle atrophy. After 14 days, the weight and CSA of the tibialis anterior muscle of denervated mice injected with SKP-SC-EVs or PBS were measured. An obvious decrease in muscle size was observed in the denervated mice after injection of PBS, whereas muscle atrophy was relatively mild after injection of SKP-SC-EVs (Figure 6B). Moreover, the wet weight ratio in the denervated PBS treatment group was significantly lower than that in the sham operation group, whereas the wet weight ratio of the SKP-SC-EVs treatment group was significantly higher than that of the denervated PBS treatment group (Figure 6C). Laminin immunofluorescence staining showed that the muscular CSA in the SKP-SC-EVs treatment group was significantly higher than that in the denervated PBS treatment group (Figure 6D,E). Therefore, SKP-SC-EVs treatment increases the mass and CSA of the tibialis anterior muscle and significantly blocks the loss of muscle in denervated mice. These findings further confirm the protective effect of SKP-SC-EVs on denervated muscle atrophy.

### 3.7. SKP-SC-EVs Inhibit Oxidative Stress and Inflammation during Denervated Muscle Atrophy

**Figure 7:**
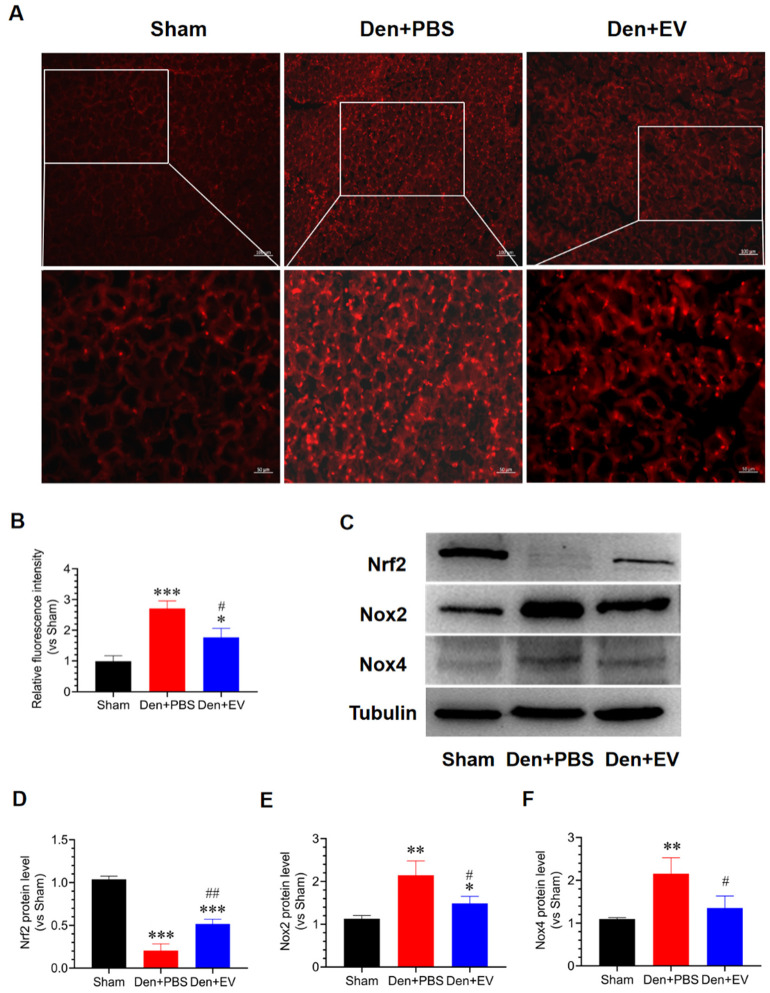
SKP-SC-EVs inhibit oxidative stress of the denervated skeletal muscle. (**A**) DHE (red) staining of the tibialis anterior muscle fiber section. Scale bar = 100 μm (low magnification); Scale bar = 50 μm (high magnification). (**B**) Relative quantification of DHE fluorescence intensity; *n* = 5, * *p* < 0.05 and *** *p* < 0.001 vs. Sham group; # *p* < 0.05 vs. Den + PBS group. (**C**) Representative western blot detection of the levels of Nrf2, Nox2, and Nox4 in the tibialis anterior muscle in different treatment groups. (**D**–**F**) Histogram of the relative levels of Nrf2, Nox2, and Nox4 proteins. *n* = 3, * *p* < 0.05, ** *p* < 0.01, and *** *p* < 0.001 vs. Sham group; # *p* < 0.05 and ## *p* < 0.01 vs. Den + PBS group. The sham group was injected with PBS; the Den group was injected with PBS containing SKP-SC-EVs (5 × 10^10^ particles) (Den + EV group), and; the control PBS group (Den + PBS group) was injected with PBS. The tibialis anterior muscle samples were collected 14 days after treatment. Den, denervation; DHE, dihydroethidium; EVs, extracellular vesicles; SCs, Schwann cells; SKP, skin-derived precursors.

To evaluate whether SKP-SC-EVs inhibit oxidative stress and inflammation during denervated muscle atrophy, we detected the levels of ROS and inflammatory factors in the denervated tibialis anterior muscle. DHE staining results showed that the level of ROS in the denervated tibialis anterior muscle was significantly increased, and this increase was significantly inhibited by SKP-SC-EVs treatment (Figure 7A,B). The western blot assay indicated that the abundances of Nox2 and Nox4 proteins were significantly induced, and the abundance of Nrf2 protein was significantly reduced in the denervated tibialis anterior muscle. SKP-SC-EVs significantly reversed the levels of Nox2, Nox4, and Nrf2 in the denervated tibialis anterior muscle (Figure 7C–F). These results indicated that SKP-SC-EVs can inhibit oxidative stress responses that occur during denervated muscle atrophy.

**Figure 8:**
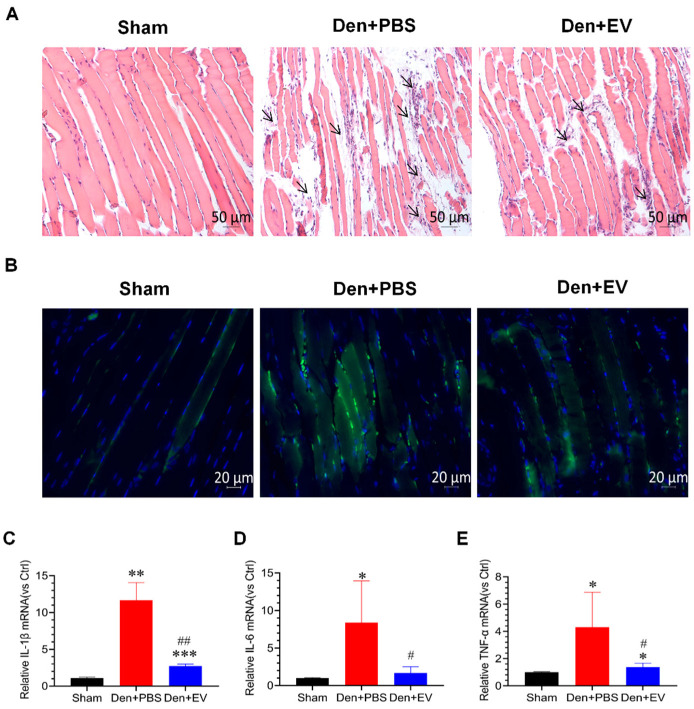
SKP-SC-EVs inhibit inflammation in the denervated skeletal muscle. (**A**) Hematoxylin-eosin staining of skeletal muscle. Black arrows indicate inflammatory cells. Scale bar = 50 μm. (**B**) CD68 immunofluorescence staining (green indicates CD68 positive signals). Scale bar = 20 μm. (**C**–**E**) qPCR analysis of the expression of inflammatory factors IL-1β, IL-6, and TNF-α in the tibialis anterior muscle. *n* = 3, * *p* < 0.05, ** *p* < 0.01, and *** *p* < 0.001 vs. Sham group; # *p* < 0.05 and ## *p* < 0.01 vs. Den + PBS group. The sham group was injected with PBS; the Den group was injected with PBS containing SKP-SC-EVs (5 × 10^10^ particles) (Den + EV group), and; the control PBS group (Den + PBS group) was injected with PBS. The tibialis anterior muscle samples were collected 14 days after treatment. Den, denervation; EVs, extracellular vesicles; TNF-α, tumor necrosis factor-α.

This study also confirmed that inflammation is significantly activated during denervated muscle atrophy. Hematoxylin-eosin staining results showed that inflammatory cells appeared in the interstitium of myofibers in the denervated PBS treatment group after denervation, which showed small nuclei and clusters, whereas the inflammatory cells in the SKP-SC-EVs-treated group were significantly reduced after denervation (Figure 8A). Further analysis by CD68 immunofluorescence staining showed that a large number of positive signals appeared in the muscular interstitium of the denervated PBS treatment group, and SKP-SC-EVs treatment significantly inhibited the production of positive signals (Figure 8B). In addition, the increased levels of inflammatory cytokines mRNA, such as IL-1β, IL-6, and TNF-α, indicated inflammation during muscle atrophy, and SKP-SC-EVs treatment significantly reduced the levels of these inflammatory cytokines (Figure 8C–E). In summary, SKP-SC-EVs can significantly inhibit inflammatory responses during denervated muscle atrophy.

### 3.8. SKP-SC-EVs Inhibit the UPS Pathway during Denervated Muscle Atrophy

**Figure 9:**
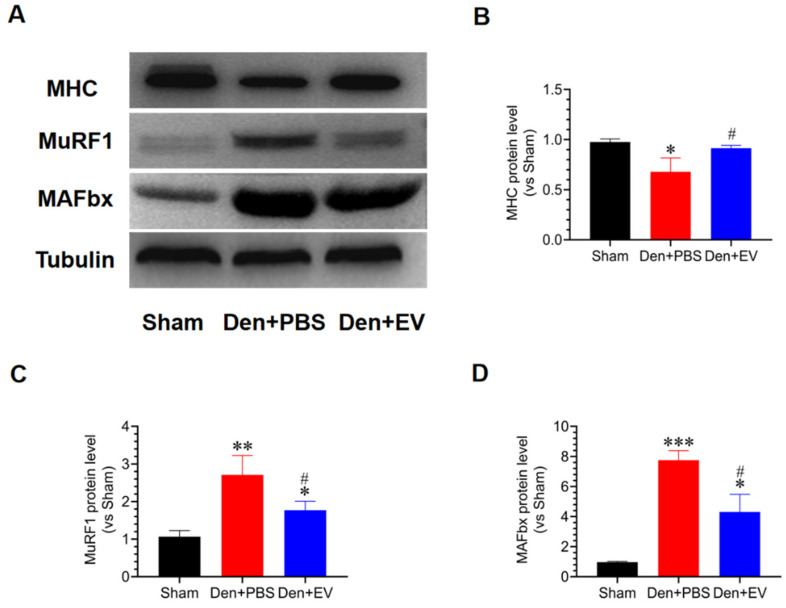
SKP-SC-EVs inhibit ubiquitinated proteolysis during denervated muscle atrophy. (**A**) Representative western blot detection of MHC, MuRF1, and MAFbx in different treatment groups. (**B**–**D**) Histogram showing the relative abundances of MHC, MuRF1, and MAFbx in the tibialis anterior muscle after denervation. *n* = 3, * *p* < 0.05, ** *p* < 0.01, and *** *p* < 0.001 vs. Sham group; # *p* < 0.05 vs. Den + PBS group. The sham group was injected with PBS; the Den group was injected with PBS containing SKP-SC-EVs (5 × 10^10^ particles) (Den + EV group), and; the control PBS group (Den + PBS group) was injected with PBS. The tibialis anterior muscle samples were collected 14 days after treatment. Den, denervation; EVs, extracellular vesicles; IL, interleukin; SCs, Schwann cells; SKP, skin-derived precursors; TNF-α, tumor necrosis factor-α.

To evaluate the effects of SKP-SC-EVs on the UPS during denervated muscle atrophy, western blot analysis was conducted, which showed that protein degradation occurs during denervated muscle atrophy. Compared with the control group, the expression of the MHC protein was significantly reduced in the denervated PBS treatment group; however, the expression of two key enzymes in the UPS pathway, MuRF1 and MAFbx, increased. Interestingly, SKP-SC-EVs treatment significantly inhibited the increase in the expression of muscle-specific E3 ubiquitin ligases, MAFbx and MuRF1, thereby inhibiting the hydrolysis of MHC (Figure 9). These findings indicated that SKP-SC-EVs can inhibit the UPS pathway during denervated muscle atrophy.

### 3.9. SKP-SC-EVs Inhibit Mitochondrial Autophagy during Denervated Muscle Atrophy

**Figure 10:**
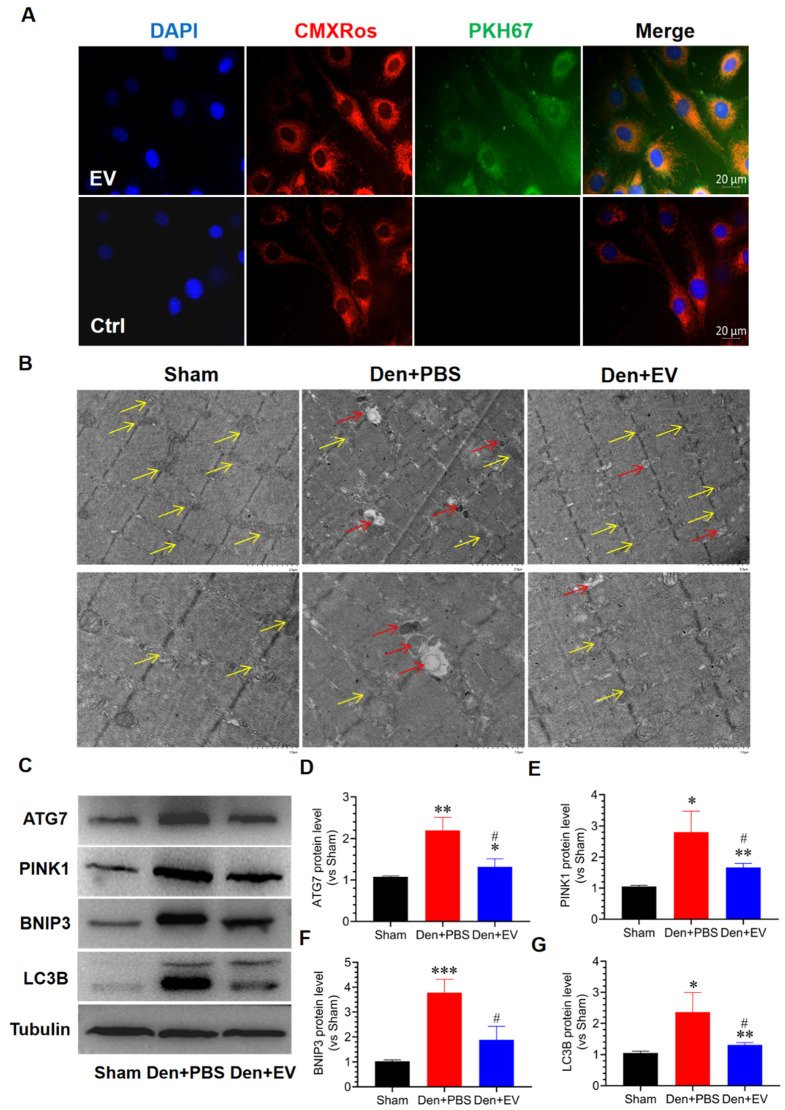
SKP-SC-EVs inhibit denervation-induced skeletal muscle autophagy. (**A**) Co-localization of SKP-SC-EVs and mitochondria: PKH67-labeled SKP-SC-EVs (green), CMXRos-labeled mitochondria (red), and nuclei labeled with DAPI (blue). Scale bar = 20 μm. The EV group was given SKP-SC-EVs, but the Ctrl group was not. (**B**) Electron microscope observation of the tibialis anterior muscle. The lower image shows an enlarged part of the upper image. The yellow arrow indicates normal mitochondria, and the red arrow indicates mitochondrial autophagy. (**C**) Representative western blot analysis of autophagy-related proteins ATG7, PINK1, BNIP3, and LC3B in the tibialis anterior muscle in different treatment groups. (**D**–**G**) Histogram of the relative expressions of ATG7, PINK1, BNIP3, and LC3B in the tibialis anterior muscle in different treatment groups. *n* = 3, * *p* < 0.05, ** *p* < 0.01, and *** *p* < 0.001 vs. Sham group; # *p* < 0.05 vs. Den + PBS group. The sham group was injected with PBS; the Den group was injected with PBS containing SKP-SC-EVs (5 × 10^10^ particles) (Den + EV group), and; the control PBS group (Den + PBS group) was injected with PBS. The tibialis anterior muscle samples were collected 14 days after treatment. Den, denervation; EVs, extracellular vesicles; SCs, Schwann cells; SKP, skin-derived precursors.

Skeletal muscle is a motor-effect organ that requires energy from mitochondria, and mitochondrial dysfunction is an important feature of skeletal muscle atrophy [51]. To understand whether SKP-SC-EVs inhibit mitochondrial autophagy and repair mitochondrial function during denervated skeletal muscle atrophy, a Mito Tracker Red CMXRos fluorescent probe and PKH67 were used to label mitochondria in C2C12 cells and SKP-SC-EVs, respectively. We found that SKP-SC-EVs and mitochondria were co-localized by co-culturing C2C12 cells and SKP-SC-EVs. Therefore, an interaction or signal exchange may exist between them (Figure 10A). Furthermore, we analyzed whether SKP-SC-EVs have a protective effect on mitochondria by observing the ultrastructure of the tibialis anterior muscle under transmission electron microscopy. We found mitochondrial vacuolation and autophagy in the denervated skeletal muscle after treatment with PBS. Moreover, myofilaments and sarcomeres were not neatly arranged, and mitochondria were arranged disorderedly. Thus, SKP-SC-EVs treatment could significantly inhibit mitochondrial vacuolation and autophagy caused by the denervation of skeletal muscle (Figure 10B). We further explored the effect of SKP-SC-EVs on autophagy-related proteins (PINK1, BNIP3, LC3B, and ATG7) and found that the expression of PINK1, BNIP3, LC3B, and ATG7 in the skeletal muscle of the denervated PBS treatment group was significantly increased, whereas the expression of mitochondrial autophagy-related proteins in the skeletal muscle of the SKP-SC-EV treatment group was significantly reduced (Figure 10C–G). These findings indicated that SKP-SC-EVs may alleviate mitochondrial autophagy or vacuolation, effectively reduce mitochondrial damage, and inhibit the activity of autophagy-lysosomal pathways by inhibiting the expression of mitochondrial autophagy-related proteins, thereby alleviating denervated muscle atrophy.

### 3.10. SKP-SC-EVs Improve Microvascular Blood Flow in the Denervated Skeletal Muscle

**Figure 11:**
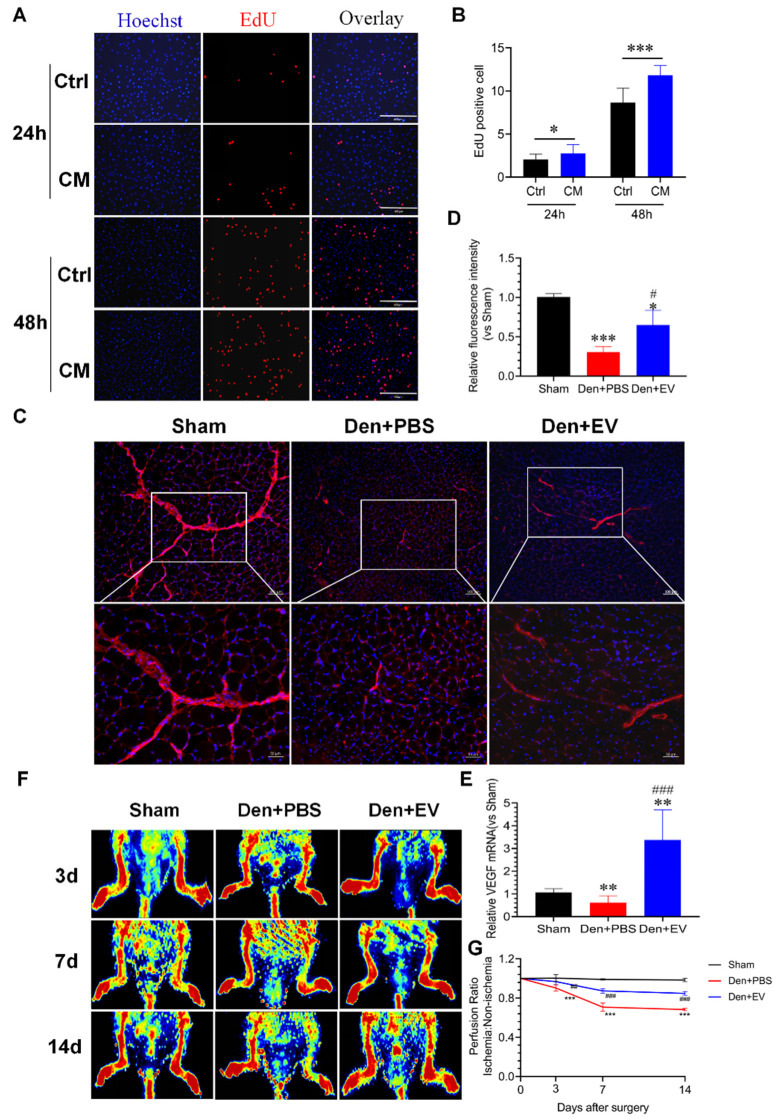
SKP-SC-EVs promote the proliferation of HUVEC cells and improve blood flow reperfusion in the denervated skeletal muscle. (**A**) EdU staining of HUVEC cells. Scale bar = 400 μm. (**B**) Histogram showing the effect of the SKP-SC conditioned medium on the proliferation of HUVEC cells. *n* = 4, * *p* < 0.05 and *** *p* < 0.001 vs. Control group. (**C**) The effect of SKP-SC-EVs on the micro-blood flow in denervated skeletal muscles. Red indicates CD31 positive signals, and blue indicates the DAPI-labeled nucleus. Scale bar = 100 μm (low magnification); scale bar = 50 μm (high magnification). (**D**) Histogram showing the relative fluorescence intensity of the CD31 positive signal. *n* = 5, * *p* < 0.05 and *** *p* < 0.001 vs. Sham group; # *p* < 0.05 vs. Den + PBS group. (**E**) qPCR analysis of the expression of VEGF in the tibialis anterior muscle. *n* = 3, ** *p* < 0.01 vs. Sham group; ### *p* < 0.001 vs. Den + PBS group (**F**) Laser Doppler blood flow imaging analysis of the influence of SKP-SC-EVs on the micro-blood flow in the denervated skeletal muscle. (**G**) Histogram showing the blood perfusion ratio of the ischemic lower limb and the contralateral non-ischemic lower limb at different times after denervation. *n* = 6, *** *p* < 0.001 vs. Sham group; ## *p* < 0.01 and ### *p* < 0.001 vs. Den + PBS group. The sham group was injected with PBS; the Den group was injected with PBS containing SKP-SC-EVs (5 × 10^10^ particles) (Den + EV group), and; the control PBS group (Den + PBS group) was injected with PBS. The tibialis anterior muscle samples were collected 14 days after treatment. Den, denervation; EVs, extracellular vesicles; HUVEC, human umbilical vein endothelial cell; SCs, Schwann cells; SKP, skin-derived precursors.

The main cause of muscle atrophy after denervation is the loss in nutrient supply and voluntary contraction in the denervated muscle, which results in the inability to pump fresh blood, leading to relative ischemia and hypoxia. Therefore, ROS and inflammatory factors are produced, and capillary damage occurs, which in turn, leads to the occlusion of the capillary bed. This further aggravates ischemia and hypoxia and activates inflammation, thereby activating the proteolytic pathway, which causes proteolysis and muscle atrophy [4]. Our previous study found that nerve grafts constructed from SKP-SC-EVs promote angiogenesis, improve the microenvironment, and promote the expression of angiogenesis-related factors, whilst also promoting nerve regeneration [41]. Therefore, we further explored the potential effect of SKP-SC-EVs on blood flow reperfusion during denervated skeletal muscle atrophy. To this end, we investigated the effects of SKP-SC-EVs on the proliferation of HUVECs in vitro. The EdU staining analysis showed that a 24 or 48 h co-culture of SKP-SCs and HUVECs significantly promotes the proliferation of HUVECs in a dose-dependent manner. This also suggested that EVs secreted by SKP-SCs promote the proliferation of HUVECs co-cultured with SKP-SCs (Figure 11A,B). In summary, SKP-SC-EVs may have a pro-angiogenesis effect.

We further explored the effect of SKP-SC-EVs on the microvessels in skeletal muscle after denervation. CD31 immunofluorescence staining revealed that the microvessels in the mouse tibialis anterior muscle were coherent and thick in the sham operation group, intermittent in the denervated PBS treatment group, and continuous and thickened in the SKP-SC-EVs treatment group (Figure 11C,D). The qPCR analysis showed that compared with the control group, the expression of the pro-angiogenic factor VEGF in the tibialis anterior muscle was reduced 14 days after denervated muscle atrophy. However, SKP-SC-EVs treatment significantly increased the expression of VEGF (Figure 11E). Subsequently, laser Doppler blood flow imaging analysis revealed that there was a gradual decline in the blood flow of the lower limbs on the nerve injury side of the denervated PBS-treated mice 3, 7, and 14 days after denervation, whereas SKP-SC-EVs treatment induced a significant improvement in the blood flow in the lower limbs on the nerve injury side (Figure 11F,G). Thus, SKP-SC-EVs may improve blood flow in the denervated skeletal muscle by enhancing the proliferation of vascular endothelial cells.

## 4. Discussion

**Figure 12:**
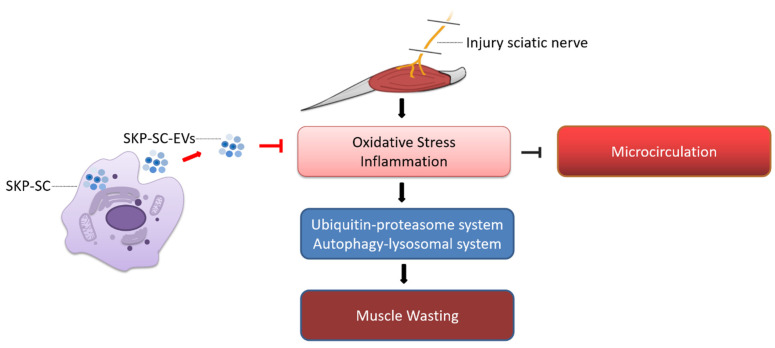
Schematic diagram illustrating how SKP-SC-EVs may alleviate denervation-induced decrease in the blood flow of target muscles by inhibiting oxidative stress and inflammation, improving microcirculation, and inactivating the ubiquitinated proteolytic and autophagy-lysosomal pathways, thereby alleviating denervated skeletal muscle atrophy.

This study demonstrated for the first time that SKP-SC-EVs are internalized and taken up by muscle cells, thereby delaying skeletal muscle atrophy caused by denervation. Our findings indicated that SKP-SC-EVs inhibit denervation-induced oxidative stress and inflammation in skeletal muscle, promote the proliferation of vascular endothelial cells, improve microcirculation, inhibit denervation-induced activation of the UPS and ALS pathways and mitochondrial autophagy, and significantly relieve denervated muscle atrophy (Figure 12).

An appropriate level of active oxygen can effectively defend against pathogens. However, the continuous overproduction of ROS causes cell and tissue damage [52]. Our previous study found that ROS and inflammation play a key role in denervated muscle atrophy. Overproduction of ROS and pro-inflammatory cytokines are two important molecular mechanisms involved in muscle atrophy, which suggests that anti-inflammation or anti-oxidation therapy may be an effective treatment for muscle atrophy [4]. Recent studies have shown that EVs derived from stem/progenitor cells have similar effects to cell therapy, without the need for the large number of cells that are required for stem cell therapy. EVs can overcome the low survival rate associated with cells used in cell therapy, and effective targeting and therapeutic safety can be ensured because EVs can be more quickly internalized [53]. EVs are active substances that can be secreted by almost all cells and act as nano-level messengers that transmit biological signals, mediate cell-to-cell communication, and participate in a wide range of biological processes. EVs from therapeutic cells can strengthen tissue regeneration and serve as a potential alternative to stem cell therapy [54,55]. Therefore, EVs have become highly attractive and may be a candidate drug for numerous diseases. Previous studies have shown that SKP-SCs have an effect similar to that of primary SCs, such as the promotion of the functional recovery of nerve cells after treatment. SKP-SC-EVs have potential comparable to that of SKP-SCs and can significantly enhance the regeneration and recovery of damaged nerve cells [40,56]. In addition, we sequenced and analyzed SKP-SC-EVs in our previous study and found that they contained abundant miRNAs with antioxidant and anti-inflammatory effects, including miR-30a-5p, miR-22, miRNA-23a, and miR-27a/b. Therefore, we speculate that SKP-SC-EVs may also have good application prospects in the treatment of target muscle denervation.

In this study, the SKP-SCs expanded in vitro showed good proliferation ability and stability. After culturing for 2 days in a fetal-free bovine serum proliferation medium, the mass release of EVs from SKP-SCs was elicited, after which EVs were separated from the medium to construct a cell-free therapy platform based on SKP-SC-EVs. This is an important basis for the treatment of denervated muscle atrophy using cell-free therapy. We applied SKP-SC-EVs to denervated target muscles and observed and evaluated the muscle wet weight ratio and muscle fiber CSA in different treatment groups to detect the inhibitory effect of SKP-SC-EVs on oxidative stress and inflammation as well as the improvement effect on microcirculation. We then analyzed the influence of SKP-SC-EVs on the proteolytic pathway and mitochondrial autophagy and evaluated the protective effect and mechanism of SKP-SC-EVs on denervated muscle atrophy.

The prerequisite for SKP-SC-EVs to elicit intracellular communication is the internalization of EVs by the recipient cells. Theoretically, although the contents of SKP-SC-EVs have not yet been determined, EVs can be successfully transferred into target cells, providing myriad signal molecules, which include RNA, proteins, and lipids. In this study, PKH67-labeled SKP-SC-EVs were effectively captured by C2C12 cells and mainly accumulated in the cytoplasm. In vivo studies have revealed that after local microinjection of SKP-SC-EVs into target muscles, muscle fibers can also significantly capture SKP-SC-EVs. Although the biodistribution of SKP-SC-EVs is controlled by different routes of administration, our results showed that effective uptake of SKP-SC-EVs can be ensured using multi-site local microinjection into the tibialis anterior muscle. These findings indicate that SKP-SC-EVs can be effectively delivered to muscle cells both in vivo and in vitro, and interact with target cells.

SKP-SC-EVs can dose-dependently promote the proliferation and differentiation of C2C12 cells under physiological conditions. Similar findings have also been found in EVs derived from adipose-derived mesenchymal stem cells, which promote muscle growth [57]. In another study, EVs derived from human placental mesenchymal stem cells alleviated experimental colitis by inhibiting inflammation and oxidative stress [58]. EVs derived from human umbilical cord mesenchymal stem cells reduced liver ischemia-reperfusion injury in rats by inhibiting oxidative stress and neutrophil inflammation [59]. Moreover, EVs derived from mesenchymal stem cells reduced mitochondrial damage and inflammation by stabilizing mitochondrial DNA [60]. Based on these findings, we explored whether SKP-SC-EVs can also inhibit oxidative stress and inflammation during denervated muscle atrophy, thereby preventing muscle atrophy. We found that in vitro SKP-SC-EVs treatment reduced the levels of ROS and inflammatory factors in myotubes induced by nutrient deprivation, which indicated that SKP-SC-EVs can protect nutrition deprivation-induced myotube atrophy, at least in part, by inhibiting oxidative stress and inflammation. In vivo studies indicated that SKP-SC-EVs block the loss of muscle mass induced by denervation, which leads to a reduction in muscle atrophy. In addition, SKP-SC-EVs inhibited the production of ROS and inflammatory cytokines in denervated skeletal muscle, which suggested that SKP-SC-EVs inhibit oxidative stress and inflammation in denervated skeletal muscle.

Muscle atrophy causes a loss in muscular movements accompanied by a lack of fresh blood supply, which leads to significant secondary injuries, such as ischemia and hypoxia, local microvascular damage, vascular stenosis, or even occlusion. Maintaining the homeostasis of the regenerative microenvironment is essential for muscle growth and development. Based on the EVs delivery system, we constructed an endogenous beneficial microenvironment and activated the protection mechanism underpinning tissue regeneration, which is a promising method [61,62]. Increasing evidence has shown that EVs actively regulate angiogenesis in vascular endothelial cells by acting on blood vessel formation [63]. Stimulated by platelet-derived growth factor, adipose tissue-derived mesenchymal stem cells (ASCs) can release EVs that are rich in anti-inflammatory and immunoregulatory factors and induce an increase in the production of IL-10 and TGF-β1, thereby protecting muscles against acute ischemia [64]. EVs derived from mesenchymal stem cells can activate VEGF receptors, which activate the pro-angiogenic pathways of SRC, AKT, and ERK, and promote the recovery of the ischemic hind limbs [65]. These findings suggest that SKP-SC-EVs have the potential to repair secondary ischemic/hypoxic damage in muscle cells caused by denervation. In this study, microcirculation in the denervated tibialis anterior muscle was impaired, and blood flow decreased significantly; however, SKP-SC-EVs treatment improved microcirculation and blood flow in the denervated tibialis anterior muscle. After treatment with SKP-SC-EVs, the proliferation ability of vascular endothelial cells in skeletal muscle was significantly increased; moreover, the microcirculation in the denervated skeletal muscle was improved. The recovery of damaged blood vessels may be due to the SKP-SC-EVs-mediated inhibition of oxidative stress and inflammation, which improves the proliferation of vascular endothelial cells, thereby improving microcirculation, further reducing oxidative stress and inflammation, and eventually alleviating denervated muscle atrophy.

A significant increase in proteolytic activity can directly cause skeletal muscle atrophy. Previous studies have shown that skeletal muscle atrophy is accompanied by significant activation of the proteolytic UPS and ALS pathways [13,14]. Moreover, oxidative stress and inflammation can trigger proteolysis by activating the UPS and ALS pathways [66]. In this study, SKP-SC-EVs hindered the loss of muscle mass induced by denervation and the reduction in muscle atrophy. Furthermore, SKP-SC-EVs inhibited the increase in expression of MAFbx and MuRF1 in denervated target muscles, which are two key enzymes of the UPS pathway for proteolysis, which indicated that SKP-SC-EVs inhibit the activation of the UPS pathway through the inhibition of oxidative stress and inflammation. In addition, ROS stimulation damages intracellular mitochondria, which triggers the formation of autophagic vacuoles. Mitochondrial phagocytosis is also crucial to skeletal muscle function. Damaged mitochondrial phagocytosis leads to the accumulation of damaged mitochondria and affects muscle mass [49]. In this study, denervation of skeletal muscle significantly induced mitochondrial autophagy, and SKP-SC-EVs inhibited the production of autophagosomes or autophagic vacuoles, accompanied by a decrease in the expression of mitochondrial autophagy-related proteins. These results are consistent with the findings of other studies. Adipose-derived mesenchymal stem cells secrete EVs containing miR-25-3p to trigger neuroprotection by improving autophagy [67]. In patients with COPD, the upregulated content of miR-210 in EVs derived from human bronchial epithelial cells directly regulates autophagy by targeting ATG7 and inhibits the differentiation of myofibroblasts that are promoted by autophagy [68]. Therefore, SKP-SC-EVs may reduce mitochondrial autophagy in denervated skeletal muscle by inhibiting oxidative stress and inflammation.

Our previous studies revealed that denervated skeletal muscles lose their contraction ability, which results in the inability to pump out venous blood, and arterial blood is unable to perfuse the skeletal muscles, causing ischemia and hypoxia. Consequently, a large amount of ROS is produced, which causes oxidative stress damage. If the damage is not relieved promptly, an inflammatory response occurs, which further damages muscle cells and capillaries, aggravates oxidative stress damage and inflammation, and induces the extensive activation of the proteolytic pathways [4]. We were the first to use SKP-SC-EVs to treat denervated muscle atrophy and found that they promoted the proliferation and differentiation of myoblasts and offered a good therapeutic effect on myotube atrophy caused by nutrient deprivation or denervation. Such a therapeutic effect may be attributed to the fact that SKP-SC-EVs can inhibit denervation-induced oxidative stress and inflammation and improve microcirculation and blood perfusion in the denervated skeletal muscle, thereby inhibiting the activation of proteolytic pathways and ultimately delaying denervated muscle atrophy (Figure 12). This work established an experimental basis for exploring the complex mechanism by which SKP-SCs-EVs improve denervated muscle atrophy and for the preclinical translational test of SKP-SC-EVs.

A valuable avenue of research is to develop effective treatment strategies based on the EVs platform. Diverse EVs biogenesis mechanisms and the complex EVs contents contribute to the different functions of EVs from different cell sources; thus, standardization strategies of different EV drugs warrant exploration in future work. In addition, the following aspects should be considered when optimal practical models for the therapeutic use of EVs have been developed: cell culture conditions, EVs extraction methods, yield, and purity. Finally, clarifying the complex protective mechanism underlying muscle growth remains a challenge, and the interactions involve varied and complex multicellular responses. Thus, it is necessary to further verify the therapeutic potential of SKP-SC-EVs for denervated muscle atrophy. Therefore, we plan to employ a variety of muscle atrophy models, conduct more functional and safety tests, and gain a better understanding of the contents of EVs to clarify the potential and mechanism of the biologically active ingredients contained in SKP-SC-EVs that enable the delaying or even reversing of denervated muscle atrophy.

## 5. Conclusions

This study proposed, for the first time, that SKP-SC-EVs treatment effectively delays muscle atrophy in mice caused by sciatic nerve transection. Antioxidation and anti-inflammation therapy may be effective for treating denervated muscle atrophy. SKP-SC-EVs have significant therapeutic potential and may be a potential drug candidate for the prevention and treatment of muscle atrophy. Our results further emphasize the value of determining the molecular mechanisms in follow-up work.

## Figures and Tables

**Table 1:** List of Primer Sequences.

Gene (Accession Number)		Primer Sequences (5′→3′)
Nrf2 (18024)	Forward	TAGATGACCATGAGTCGCTTGC
	Reverse	GCCAAACTTGCTCCATGTCC
Nox2 (13058)	Forward	TGAATGCCAGAGTCGGGATTT
	Reverse	CGAGTCACGGCCACATACA
Nox4 (50490)	Forward	TGCCTGCTCATTTGGCTGT
	Reverse	CCGGCACATAGGTAAAAGGATG
NQO1 (18104)	Forward	AGGATGGGAGGTACTCGAATC
	Reverse	TGCTAGAGATGACTCGGAAGG
IL-6 (16193)	Forward	CTGCAAGAGACTTCCATCCAG
	Reverse	AGTGGTATAGACAGGTCTGTTGG
IL-1β (16176)	Forward	GAAATGCCACCTTTTGACAGTG
	Reverse	TGGATGCTCTCATCAGGACAG
TNF-α (21926)	Forward	CTGAACTTCGGGGTGATCGG
	Reverse	GGCTTGTCACTCGAATTTTGAGA
VEGF (22339)	Forward	GCTGCTGTAACGATGAAG
	Reverse	ATCTGCTGTGCTGTAGGA
18s (19791)	Forward	CAGCCACCCGAGATTGAGCA
	Reverse	TAGTAGCGACGGGCGGTGT

## Data Availability

Data is contained within the article.

## Supplementary Materials

The following supporting information can be downloaded at: https://www.mdpi.com/article/10.3390/antiox11010066/s1, Supplementary Figure S1: Identification of SKP-SCs; Supplementary Figure S2: SKP-SC-EV biological activity verification.
