# Supplementary material for: SKP-SC-EVs Mitigate Denervated Muscle Atrophy by Inhibiting Oxidative Stress and Inflammation and Improving Microcirculation
Source: Antioxidants (Basel). 2021 Dec 28;11(1):66. doi: 10.3390/antiox11010066 (PMC8772917; doi:10.3390/antiox11010066)
Supplement: Supplementary file 1 [file antioxidants-11-00066-s001.zip › antioxidants-1494568-supplementary.pdf]

<u>**Supplemental Figures**</u>

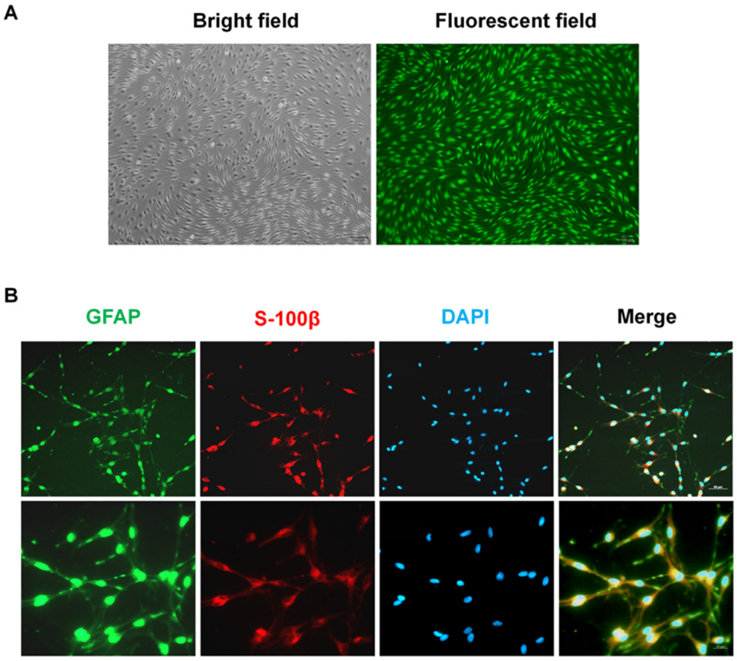

Supplementary Figure S1. Identification of SKP-SCs. (**A**) The SCs from SKP were spindle-shaped and arranged in parallel. Scale bar = 100 μm. (**B**) SCs derived from SKP were positive for GFAP (green) and S-100β (red), and the nucleus was labeled with DAPI (blue). Scale bar = 50 μm (low magnification); scale bar = 20 μm (high magnification). SCs, Schwann cells; SKP, skin-derived precursors.

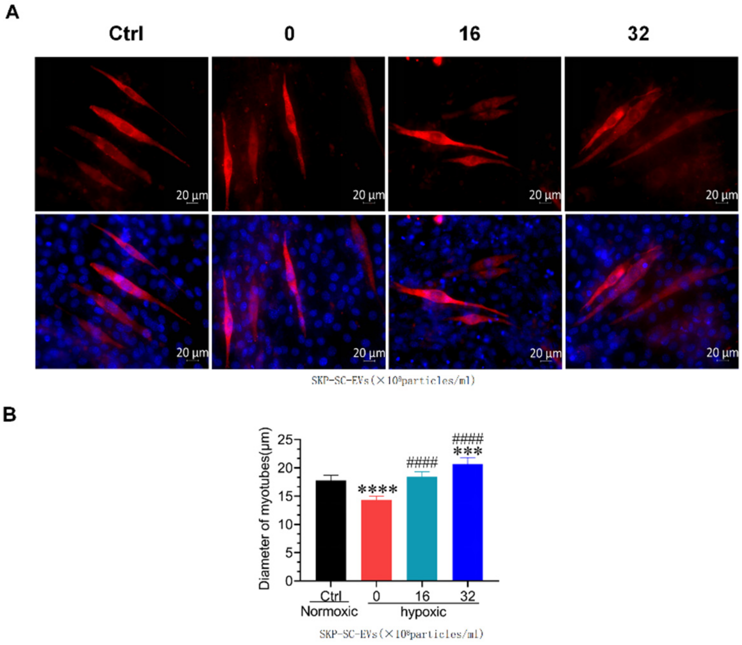

Supplementary Figure S2. SKP-SC-EV biological activity verification. (**A**) MHC immunofluorescence staining of C2C12 myotubes. Myotubes after hypoxia were treated with different doses of SKP-SC-EVs. After 12 hours of hypoxia, we observed the effects of SKP-SC-EVs on

C2C12 myotubes. Scale bar = 20 μm. (**B**) Histogram indicating the change in myotube diameter in different-dose treatment groups. ***$p < 0.001$ and ****$p < 0.0001$ vs. control (Ctrl) group; ####$p < 0.0001$ vs. 0 group. EVs, extracellular vesicles. SCs, Schwann cells; SKP, skin-derived precursors.
